# Power distance and mentor-protégé relationship quality as moderators of the relationship between informal mentoring and burnout: evidence from China

**DOI:** 10.1186/1752-4458-8-51

**Published:** 2014-12-11

**Authors:** Jing Qian, Zhuo Han, Haiwan Wang, Xiaoyan Li, Qiuyue Wang

**Affiliations:** Business School, Beijing Normal University, No. 19 Xinjiekou Outer Street, Beijing, 100875 China; School of Psychology, Beijing Normal University, No. 19 Xinjiekou Outer Street, Beijing, 100875C China; School of Business, Jiangxi Normal University, No. 99 Ziyang Street, Nanchang, 100875 China

**Keywords:** Burnout, Mentoring, Power distance, Mentor-protégé relationship quality

## Abstract

**Background:**

The topic of how to prevent and reduce burnout has drawn great attention from researchers and practitioners in recent years. However, we know little about how mentoring as a form of social support exerts influence on employee burnout.

**Aim:**

This study aims to examine the contingency side of the mentoring-burnout relationship by addressing the exploratory question of whether individual differences in power distance and relationship quality play important roles in mentoring effectiveness in terms of reducing a protégé’s burnout level.

**Methods:**

A total of 210 employees from a technology communications company completed the survey questionnaire.

**Results:**

(1) A protégés’ power distance moderates the negative relationship between mentoring and burnout in such a way that the relationship is stronger for protégés who are lower rather than higher in power distance; (2) mentor-protégé relationship quality moderates the negative relationship between mentoring and burnout in such a way that the relationship is stronger when the relationship quality is higher rather than lower.

**Conclusions:**

In sum, our results highlight the importance of studying the contingency side of mentoring effects on protégé burnout. Our findings suggest that the individuals’ different cultural values of power distance and mentor-protégé relationship quality are the boundary conditions for the mentoring-burnout relationship. We therefore suggest that research on mentoring-burnout will be advanced by considering the role of the moderating process.

## Background

Burnout refers to a psychological response to chronic work stress combining emotional exhaustion, depersonalization and reduced professional efficacy [1, 2]. Given the high costs and negative consequences associated with burnout such as reduced productivity, performance and commitment as well as increased turnover, absenteeism and organizational health care costs, the topic of how to prevent and reduce burnout has drawn great attention for researchers and practitioners in recent years (e.g., [3, 4]). The resulting efforts have shown that the individual employees’ personal characteristics such as sense of control and personality, and/or the contextual factors such as leader behaviors and performance pressure are the antecedents of burnout at work (e.g., [4–6]).

Workplace informal mentoring refers to a developmentally oriented relationship voluntarily initiated and maintained between a less experienced employee (the protégé) and a more experienced employee (the mentor) where the goal is the personal and professional development of the protégé [7]. Our study focuses on informal mentoring as previous studies have suggested that compared with formal mentoring, informal mentoring provides greater psychological support and has longer term effects on protégés (e.g., [8, 9]). According to social support theory, individuals tend to seek out and count on supportive relationships to prevent and reduce burnout [10]. As a form of social support, it therefore seems straightforward that mentoring can help employees reduce job burnout. To the best of our knowledge, only two empirical studies have examined the relationship between mentoring and burnout. Thomas and Lankau’s [11] findings suggest that there is a negative relationship between mentoring and burnout. In the study conducted by Eby, Butts, Durley and Ragins [12], however, the negative relationship between mentoring and burnout (they used only the emotional exhaustion dimension) is not significant, indicating that the effect of informal mentoring on burnout is not yet sufficiently clear. There might be possible moderators. Recent research suggests that protégés may differ in their response to mentoring based on personal differences or contextual factors (e.g., [13, 14]). In this study, we examine two example moderators for the relationship between mentoring and burnout, i.e., an individual difference construct of power distance and the contextual factor construct of mentor-protégé relationship quality. By doing so, our research seeks to provide a more complete picture about the influence of informal mentoring on burnout. Specifically, we examine the moderating influence of individual cultural differences in power distance and the contextual factor of mentor-protégé relationship quality on the burnout-reduction effects of mentoring using a Chinese sample. In this study, we examine two example moderators for the relationship between mentoring and burnout, i.e., an individual difference construct of power distance and the contextual factor construct of mentor-protégé relationship quality.

Power distance, defined by Hofstede [15], is the extent to which a less powerful individual expects and accepts unequally distributed power in a social context. Employees with low power distance are less constrained by the supervisor-subordinate relationship or consider it as a mainly social support. They are more willing to explore and exploit from other social resources aside from the formal interpersonal relationships at work. In addition, they are more open-minded and inclined to respect the differences between individuals that are based on experiences and ability rather than mere position. As a result, low power distance employees may perceive more value from their informal mentors’ career-related and psychological support and the mentoring-burnout relation for them is stronger.

Previous mentoring studies have pointed out that mentor-protégé relationship quality is important for mentorship effectiveness [12, 16]. When relationship quality is high, it also implies high quality communication for both parties. Mentors are more willing to share information, their thoughts and concerns without denying, distorting, exaggerating or ignoring, while protégés are more likely to perceive mentors’ good-will, feel psychologically safe, and form higher confidence in the quality of the mentoring provided. They are more willing to share life stories with mentors, try their best to sense and internalize the mentor’s psychological and career-related support and to exploit as much as possible from the mentoring relationship. Therefore, the mentoring-burnout relationship is stronger when the relationship quality is higher.

We hypothesize that (1) protégés’ power distance moderates the negative relationship between mentoring and burnout in such a way that the relationship will be stronger for protégés who are lower rather than higher in power distance and (2) mentor-protégé relationship quality moderates the negative relationship between mentoring and burnout in such a way that the relationship is stronger when the relationship quality is higher rather than lower.

## Methods

### Participants and procedure

Participants in the current study consist of 388 full-time employees of a high-tech communications company in a major city in northern China. There are three main reasons why we chose this company. First, it is a privately owned and operated firm, which generally means that the work environment is more flexible and less uniform than state-owned enterprises (SOEs) in China, leading to more unpredictable work patterns and more sources of variance regarding employees’ level of burnout (e.g., [17, 18]). Second, this firm operates within a high-tech industry where staff turnover is high and there is a continual influx of new employees. Informal mentoring, as an important part of employee orientation and career development, plays a greater role than formal mentoring. Third, this company offered no officially sanctioned formal mentoring program at the time the study was conducted.

Survey packets were distributed in a company-wide meeting. Surveys were completed on a voluntary basis. Each packet contained an information sheet explaining the objective of the survey, along with a consent form, the survey questionnaire and a return envelope with a self-sealing closure to protect the respondents' confidentiality. Participants were instructed to complete the survey and to bring it back to the upcoming meeting two weeks later. To protect participant confidentiality, they were instructed to seal the questionnaires in the envelopes provided after finishing their questionnaires. Two short messages were sent to the participants three days after the questionnaire was distributed and one day before the second meeting to encourage participants to complete the survey and to remind them to bring it with them. A box was placed outside the meeting venue, and the participants were reminded by one of the authors to put their completed and sealed questionnaire into the box before and after the meeting.

A total of 285 surveys were returned, with a response rate of 73.5%. After eliminating 43 incomplete questionnaires and 32 questionnaires that did not report any informal mentoring, 210 respondents remained and contributed to the sample of the present study. On average, protégés were 34.4 years old (*SD* = 7.51) and male (69.0%). Most participants held a bachelor’s degree (68.6%), with the remainder reporting a polytechnic diploma or associate degree (14.3%), a graduate degree (15.7%) or high school education (1.4%). The average company tenure was 8.23 years (*SD* = 6.57). A total of 62.9% were non-supervisory employees, 31.9% were first line supervisors and 5.2% were middle managers. The average number of informal mentors reported was 1.79 (*SD* = .74). The average mentorship duration for the referred mentoring relationship was 5.5 years (*SD* = 4.17). 73.8% of the mentors were male and 33.3% of the protégés had mentored others before.

### Measures

To ensure measure equivalence in the Chinese and English versions, a translation and back-translation method was applied to verify the questionnaire in Chinese. According to Behling and Law [19], this technique is necessary because creating a translation from one language to another that maintains the conceptual equivalence is very difficult due to cultural differences. For burnout, we used a seven-point response scale, and for the others, we used a five-point response scale ranging from (1) “strongly disagree” to (5) “strongly agree”.

#### Protégé status

This section was designed to 1) screen participants to identify those who currently have informal mentors, 2) instruct those who have mentors to complete the questionnaire by filling in the following five sections, 3) instruct protégés who have more than one mentor to respond to the following five sections by referring to the most influential mentor and 4) guide non-protégés to ignore the following sections and return the questionnaire on the designated date.

Whether an employee currently had an informal mentor was determined by two items preceded by the following definition based on past mentoring studies (e.g., [20]).

A mentor is an experienced employee who serves as a role model and who provides direction, support and feedback regarding career and personal development. A mentor is also someone with influence and insight, who directly provides upward mobility and/or brings your accomplishments to the attention of people who have power in the company. A mentor can be your supervisor or anybody else in the company.

Respondents were asked to indicate whether they are currently in an informal mentoring relationship. Those without an informal mentor were coded “0”. Others who had mentor(s) were also asked to give the number of informal mentors they currently had. Protégés who reported more than one mentor were instructed to complete the questionnaire by referring to the most influential mentor.

#### Mentoring function

Noe’s [21] 21-item measure of mentoring functions was used in the present study to indicate the amount of mentoring received by respondents. Some items were reworded to fit the context of the present study (the workplace setting). For example, the original item “Mentor reduced unnecessary risks that could threaten the possibility of becoming a school principal or receiving a promotion” was changed to “My mentor reduced unnecessary risks that could threaten the possibility of becoming a manager or receiving a promotion”. The *career-related mentoring functions* subscale consists of seven items (e.g., “My mentor has shared history of his/her career with me”). The *psychological mentoring functions* subscale contains 14 items (e.g., “My mentor has conveyed empathy for the concerns and feelings I have discussed with him/her”). The Cronbach’s alpha for career and psychological mentoring functions were .89 and .94, respectively. The internal consistency reliability for the scale was .96.

#### Burnout

Maslach Burnout Inventory – General survey (16 items) was used to measure protégés' burnout [22]. This inventory has been proven a reliable and valid instrument of burnout [23, 24]. This instrument includes exhaustion (five items; e.g., ‘I feel used up at the end of a work day’), cynicism (five items; e.g., ‘I doubt the significance of my work’) and professional efficacy (six items; e.g., ‘I can effectively solve the problems that arise in my work’). High scores on exhaustion and cynicism and low scores on professional efficacy are indicative of burnout. The seven-point response scale ranged from 0 (never) to 6 (daily). The reliability estimate for the scale was .93.

#### Power distance

We used the six-item scale developed by Dorfman and Howell [25]. Responses to the items used a five-point scale that ranged from 1 (strongly disagree) to 5 (strongly agree). Sample items include “Managers should make most decisions without consulting subordinates”, and “It is frequently necessary for a manager to use authority and power when dealing with subordinates”. The alpha reliability for this scale was .91.

#### Mentor-protégé relationship quality

Mentor-protégé relationship quality was measured with a three-item scale developed by Allen and Eby [26]. It has been previously used in mentoring research [12]. A sample item from this scale is “My mentor and I enjoy a high quality relationship”. The scale’s alpha coefficient was 80.

#### Control variables

We included nine control variables for testing the hypotheses. In keeping with other mentoring research (e.g., [27–31]), we controlled the participants’ age, gender, education, position, and tenure. Age and company tenure were measured by the number of years. Gender was coded 0 for “female” and 1 for “male”. Education was coded 1 for “high school”, 2 for “polytechnic diploma or associate”, 3 for “undergraduate” and 4 for “graduate”. The nominal variables of the employee position was coded 1 for “non-supervisory employees”, 2 for “first-level supervisor/manager” and 3 for “middle-level manager”.

We also controlled four mentorship status variables, as previous research has demonstrated that they could account for variance in mentoring received and/or mentoring outcomes (e.g., [32, 33]). The variables were number of mentors, mentorship duration (number of years), gender of mentor (0 = female, 1 = male) and protégé as mentor (0 = no, 1 = yes).

### Data analysis strategy

First, although the variables included in the current study are theoretically distinctive, we conducted a confirmatory factor analysis using AMOS 17.0 to empirically demonstrate the distinctiveness of mentoring, burnout, power distance, and relationship quality. Second, preliminary analyses evaluating the descriptive statistics and correlations among study variables, and possible group differences in study variables based on demographic characteristics were performed. Next, the two moderation models, with power distance and relationship quality as moderators on the relations between informal mentoring and burnout, were tested using SPSS MODPROBE macro, developed by Hayes and Matthes [34] for estimating the single-degree-of-freedom interactions in Ordinary Least Square (OLS) and logistic regression.

## Results

### Confirmatory factor analysis (CFA)

Table 1 demonstrates the results of the CFA that examined the distinctiveness of all the studied variables. We adopted the well-accepted procedure used by previous researchers to reduce the number of items for each construct by creating three indicators to represent each of them [35–38]. As shown in Table 1, the hypothesized 4-factor model fits the data well (χ2 = 154.26; df = 71; RMSEA = .01; CFI = .99; TLI = .99) and provided a significantly better fit than any alternative model, thus providing empirical evidence of the distinctiveness of the constructs studied. We therefore proceeded to test the hypotheses.

**Table 1:** **Results of confirmatory factor analysis for the studied variables**

Model	Factors	***χ*** ^2^	***df***	TLI	CFI	RMSEA
Null model		1126.992	77	.25	.45	.17
Baseline model	Four factors	154.26	71	.99	.99	.01
Model 1	Three factors: mentoring and power distance combined	315.63	74	.88	.91	.07
Model 2	Three factors: mentoring and relationship quality combined	737.60	74	.54	.67	.13
Model 3	Two factors: mentoring, relationship quality and power distance combined	887.27	76	.44	.59	.14

1) N = 210 with listwise deletion.

2) TLI is the Tucker-Lewis index; CFI, the comparative fit index; and RMSEA, the root-mean-square error of approximation.

### Preliminary analyses

See Table 2 for correlations among study variables, mean scores, and standard deviations. Initial analyses examined participants’ age, gender, education, position and tenure differences on all variables. Pearson’s correlational test demonstrated no age difference on any study variable. Independent samples *t*-tests demonstrated no gender difference on any study variable. One-Way ANOVA tests showed no education, position or tenure differences on any study variable, except for the effect of education difference on burnout. Post-hoc analysis indicated that people with undergraduate degree reported more job burnout than people with graduate degree [F (3, 206) = 2.93, *p* = .035].

**Table 2:** **Means, standard deviations, and correlations among study variables**

		Mean	SD	1	2	3
1.	Mentoring	4.57	.86			
2.	Burnout	2.47	.78	-.39^**^		
3.	Power distance	3.28	.72	-.11	-.05	
4.	Relationship quality	3.57	1.20	-.04	-.18^**^	-.34^**^

*Note:* **P < .01.

#### Moderation models

**Figure 1:**
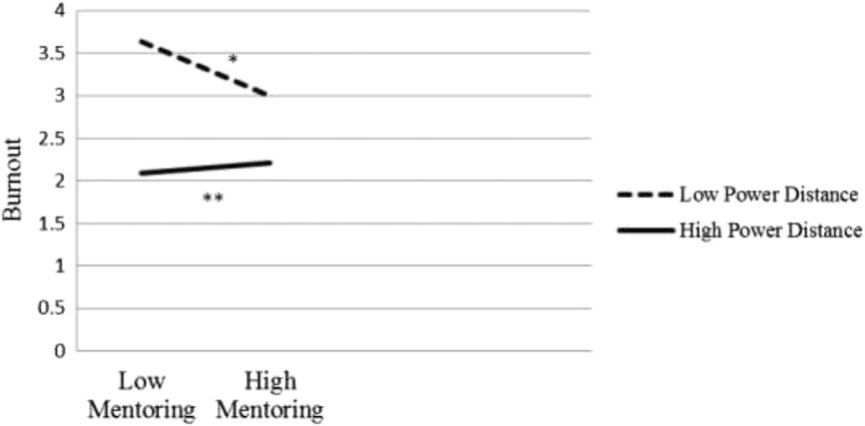
**Interaction between informal mentoring and power distance on job burnout.** *Note*: *p < .05, **p < .01.

The first moderation analysis examined whether power distance moderated links between informal mentoring and burnout. Preacher et al. [39] demonstrated that moderation is established when the independent variable and moderator significantly interact and the bootstrapped confidence interval does not contain zero. Applying the modprobe macros for moderation analysis, the conditional effect of power distance was estimated at values of one standard deviation below the mean, the mean, and one standard deviation above the mean. Demographic variables (i.e., participants’ age, gender, education, position, and tenure) and mentorship status variables were also entered in the model as controlled variables. Results showed that power distance moderated the relations between informal mentoring and job burnout (Table 3). As shown in Figure 1, the conditional effect estimates indicated that the interaction between mentoring and power distance was such that mentoring and burnout was positively associated for high power distance group, b = -.25, *p* < .000, 95% CI = (-.3874, -.1032), whereas the association was negative for low power distance group, b = -.48, *p* < .001, 95% CI = (-.6550, -.3071).

**Table 3:** **Results of ordinary least square regression analyses**

Moderator	b (SE)	t	R ^2^	F
Power distance			.16	12.61**
Mentoring	-.36 (.06)	-6.02**		
Power distance	-.07 (.07)	-.97		
Mentoring *power distance	.16 (.07)	2.21*		
Relationship quality			.21	18.50**
Mentoring	-.34 (.06)	-5.91**		
Relationship quality	-.13 (.04)	-3.23*		
Mentoring *relationship quality	.16 (.05)	3.46**		

*Note*: *p < .05, **p < .01.

**Figure 2:**
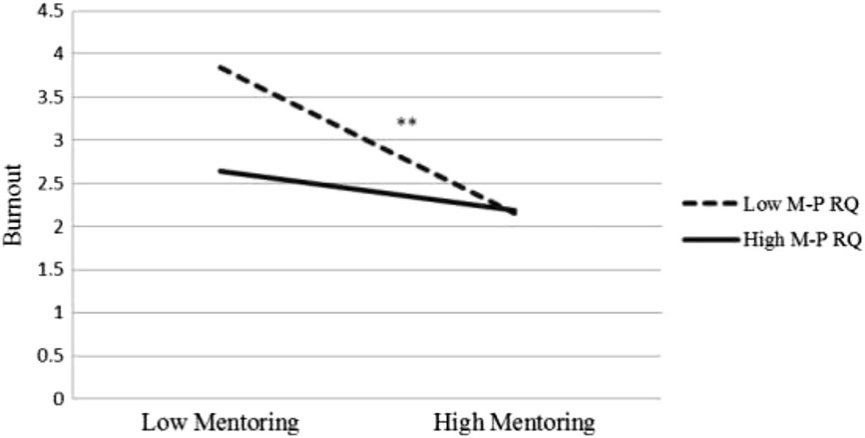
**Interaction between informal mentoring and mentor-protégé relationship quality on job burnout.** *Note*: *p < .05, **p < .01.

Similarly, the second mediation analysis examined whether mentor-protégé relationship quality moderated the relations between informal mentoring and job burnout. Applying the modprobe macros for moderation analysis, the conditional effect of mentor-protégé relationship quality was estimated at values of one standard deviation below the mean, the mean, and one standard deviation above the mean. Demographic and mentorship status variables were again controlled in the model. Results showed that mentor-protégé relationship quality moderated the links between informal mentoring and job burnout (Table 3). Specifically, as shown in Figures 1 and 2, the conditional effect estimates indicated that the interaction between mentoring and mentor-protégé relationship quality was such that mentoring and burnout was negatively and significantly associated for low mentor-protégé relationship quality group, b = -.53, *p* = .000, 95% CI = (-.6852, -.3711), whereas the association was negative but non-significant for high mentor-protégé relationship quality group, b = -.14, *p* = .073, 95% CI = (-.2996, .0134).

## Discussion

While research into mentoring has steadily grown, the contingencies under which mentoring may be related to protégé burnout remain largely unknown. To this end, we contribute to the emerging studies investigating individual differences and contextual factors in reactions to mentoring by testing whether the relationship between informal mentoring and burnout is moderated by protégés’ power distance and mentor-protégé relationship quality (e.g., [13, 14]). The results suggest that the negative relationship between mentoring and burnout was moderated by the protégés’ cultural value of power distance in such a way that the negative relationship was stronger for protégés with a lower rather than higher level of power distance. Meanwhile, the negative relationship between mentoring and burnout was also moderated by the mentor-protégé relationship quality such that the negative relationship was stronger for protégés with a higher rather than lower quality mentoring relationship.

## Theoretical implications

The results of this study provide important contributions to the literature on burnout and mentoring in three ways. First, Halbesleben and Burkley’s [3] review of burnout research suggests the importance of social support in reducing and preventing burnout and calls for more studies to address this issue. One empirical study conducted by Thomas and Lankau [11] has shown that mentoring, as a form of social support at work, could help reduce burnout in a health care setting. Our findings revealed that mentoring is conducive to protégé burnout in a high-tech communications company in China, providing further evidence that mentoring as a form of social support could prevent and/or reduce a protégé’s burnout.

Second, we examined the contingency side of the mentoring-burnout relationship by addressing the exploratory question of whether individual differences of power distance played a moderating role in mentoring effectiveness. Whereas mentoring has made considerable progress in Western countries, a recent review of the mentoring literature shows that research conducted in other cultures has lagged behind [40]. Although some researchers have conducted mentoring field studies in other cultural settings (e.g., [41, 42]), the unique influence that cultural values have on protégés has not been theorized or empirically examined.

Additionally, The reason we test our model in a Chinese setting is that the domain of organizational research is becoming more international, bringing into question the transportability of social science models from one society to another [43], and particularly to those undergoing profound transitions in institutional rules, social norms, and values [44]. A case in point is China, where workforce values are increasingly diverse, ranging from high power distance Chinese to those with low power distance with a strong Western cultural influence [45]. Therefore, a sample from China reflects the social transitions and has more variances of individual-level power distance. We examine the moderating effect of individual protégés’ power distance on the relationship between mentoring and burnout to echo the call for investigating the influences of individual diversity in values that is likely to exist in a transitional society (e.g., [44, 46]). In addition, burnout is a big issue for employees in Modern Chinese society and in recent years it has drawn great attention by the popular press (e.g., [47, 48]). More importantly, a series of programs initiated by organizations for “white-collar” employees (e.g., those work in high-tech industry) have received little rewards. All of these factors led us to conduct an empirical study in China to propose a way to reduce burnout level while proposing the potential moderators that may substantially influence the effectiveness of informal mentoring. Accordingly, our study makes a second contribution by examining the moderating effects of individual cultural value of power distance using a Chinese sample.

Third, we made a contribution by examining the moderating influence of a contextual factor of mentor-protégé relationship quality on the burnout-reduction effects of mentoring. It indicates that having a mentor is not enough, the perceived quality is of great importance in mentoring effectiveness. The popular press tends to present mentoring as an essential ingredient for employee development and organizations are trying to promote various mentoring relationships; however, very little attention has been focused on the importance of and process of building a high quality mentor-protégé relationship [16, 49, 50].

## Practical implications

This study offers some practical implications for mentoring practice. Our work suggests mentoring as a strategy to reduce and prevent employees’ burnout. A further implication stems from the results of the moderation analysis. To ensure the maximum effects of mentoring provided to employees, organizations and mentors should pay extra attention to individual differences in power distance. Special attention should be paid to those with high power distance values. For those employees, organizations could provide some complementary mentoring programs such as peer mentoring [51]. In addition, we encourage mentors not only to consider individual differences when providing mentoring functions but also to simultaneously exert an effort to enhance relationship quality. For example, mentors could build trust to improve relationship quality with protégés through the recommended strategies of perspective-taking, emotional intervention and reflection and self-corrective actions [52].

## Study limitations and future directions

Despite these findings, this study is not without limitations. First, given our cross-sectional research design, it is impossible to assert the causal relationship. Future research should incorporate longitudinal or experimental design to ascertain the causal basis of the relationship examined in this study [53]. Second, because all measures are self-reported by employees themselves, common method bias in the information obtained may be a concern. However, our analyses suggest that common method variance was not a concern here. Indeed, research suggests that common bias is less of an issue in moderated regression [54]. Although using self-report data is well-accepted in mentoring studies (e.g., [40]), we encourage future studies to adopt additional procedural remedies, such as employing a time lag between measuring independent and dependent variables. Third, in the present study, we examined two example moderators for the relationship between mentoring and burnout, i.e., an individual difference construct of power distance and the contextual factor construct of mentor-protégé relationship quality. Future studies may further explore this issue by examining other potential moderators. For example, political skill refers to “the ability to effectively understand others at work, and to use such knowledge to influence others to act in ways that enhance one’s personal or organizational objectives” ([55] pp. 127). Research by Ferris and colleagues (e.g., [56]) has described individuals possessing political skill as those who are keenly aware of their social context, are able to accurately interpret others’ behaviors and motives and to manage the social interactions. Although previous studies have acknowledged that the degree to which mentoring functions can be sensed, captured and applied depends on political skills (e.g., [57, 58]). Protégés’ political skill as an individual differences factor for mentoring effectiveness remains neglected. Future studies could therefore examine employees’ political skill as a moderator on the relationship between mentoring and burnout. Finally, in our study, we focus exclusively on informal mentoring’s influence on burnout. Participants in the current study consist of 388 full-time employees of a high-tech communications company in a major city in northern China. One of the reasons that we chose this company was that this company offered no officially sanctioned formal mentoring program at the time the study was conducted. Findings aside, we know little about how the informal mentoring-burnout relationship runs when the formal mentoring programs operate simultaneously. Future studies may investigate such an issue.

## Conclusions

In sum, our results highlight the importance of studying the contingency side of mentoring effects on protégé burnout. Our findings suggest that the individuals’ different cultural values of power distance and mentor-protégé relationship quality are the boundary conditions for the mentoring-burnout relationship. We therefore suggest that research on mentoring-burnout will be advanced by considering the role of the moderating process.
